# Primary decompressive craniectomy in poor-grade aneurysmal subarachnoid hemorrhage: long-term outcome in a single-center study and systematic review of literature

**DOI:** 10.1007/s10143-020-01383-3

**Published:** 2020-09-12

**Authors:** Simon Brandecker, Alexis Hadjiathanasiou, Tamara Kern, Patrick Schuss, Hartmut Vatter, Erdem Güresir

**Affiliations:** Department of Neurosurgery, Rheinische Friedrich-Wilhelms-University Bonn, Venusberg-Campus 1, 53127 Bonn, Germany

**Keywords:** Aneurysmal subarachnoid hemorrhage, Primary decompressive craniectomy, Long-term outcome

## Abstract

Primary decompressive craniectomy (PDC) in patients with poor-grade aneurysmal subarachnoid hemorrhage (aSAH) in order to decrease elevated intracranial pressure (ICP) is controversially discussed. The aim of this study was to analyze the effect of PDC on long-term clinical outcome in these patients in a single-center cohort and to perform a systematic review of literature. Eighty-seven consecutive poor-grade SAH patients (World Federation of Neurosurgical Societies (WFNS) grades IV and V) were analyzed between October 2012 and August 2017 at the author’s institution. PDC was performed due to clinical signs of herniation or brain swelling according to the treating surgeon. Outcome was analyzed according to the modified Rankin Scale (mRS). Literature was systematically reviewed up to August 2019, and data of poor-grade aSAH patients who underwent PDC was extracted for statistical analyses. Of 87 patients with poor-grade aSAH in the single-center cohort, 38 underwent PDC and 49 did not. Favorable outcome at 2 years post-hemorrhage did not differ significantly between the two groups (26% versus 20%). Systematic literature review revealed 9 studies: Overall, a favorable outcome could be achieved in nearly half of the patients (49%), with an overall mortality of 24% (median follow-up 11 months). Despite a worse clinical status at presentation (significantly higher rate of mydriasis and additional ICH), poor-grade aSAH patients with PDC achieve favorable outcome in a significant number of patients. Therefore, treatment and PDC should not be omitted in this severely ill patient collective. Prospective controlled studies are warranted.

## Introduction

Patients who present with a poor-grade aneurysmal subarachnoid hemorrhage (aSAH) according to the World Federation of Neurosurgical Societies (WFNS grades IV and V) often have a poor prognosis with high case fatality and disability rates [4, 7, 23, 37]. In aSAH severely brain swelling can occur primarily due to initial tissue damage with or without additional intracerebral hemorrhage and secondarily due to vasospasm/infarction. Brain swelling and elevated intracranial pressure (ICP) are known to worsen outcome following SAH [5, 10, 15]. Additionally significant predictors of unfavorable outcome in patients with poor-grade aSAH are patient age, WFNS grade V, signs of cerebral herniation, aneurysm size, and space-occupying hematoma [28]. Elderly patients suffering from aSAH should be considered for treatment despite age [32]. Decompressive craniectomy (DC) after traumatic brain injury (TBI) and space occupying stroke has been shown to reduce elevated ICP and improves outcome after ischemic stroke [1, 11, 29, 35]. To check the hypothesis that DC could improve outcome in aSAH patients similar to improve outcome in patients with space-occupying stroke, the effect of DC in poor-grade aSAH patients was reported in multiple studies: The role of aggressive surgical treatment option such as PDC remains controversial [3, 6, 8, 9, 12, 13, 16–19, 21, 22, 24–27, 30, 33, 34, 36, 38]. A systematic review and meta-analysis has shown that the effect of DC on functional outcome versus that of other interventions for refractory intracranial hypertension is still unknown [2].

We hypothesized that an early decompressive craniectomy may result in an improved neurological outcome in this severely ill subset of patients due to early control of elevated ICP, and we reported our experience with the effect of PDC on clinical outcome with long-term follow-up. A comparison was performed with a control group of poor-grade aSAH patients treated without PDC but, if necessary, receiving a secondary decompressive craniectomy due to cerebral infarction or as a treatment for increased ICP.

Additionally, we performed a systematic review of literature for PDC in poor-grade aSAH patients and therefore extracted relevant data of this subgroup of patients to clarify a possible benefit of an early decompression on clinical outcome in these patients.

## Material and methods

### Patient population

**Fig. 1:**
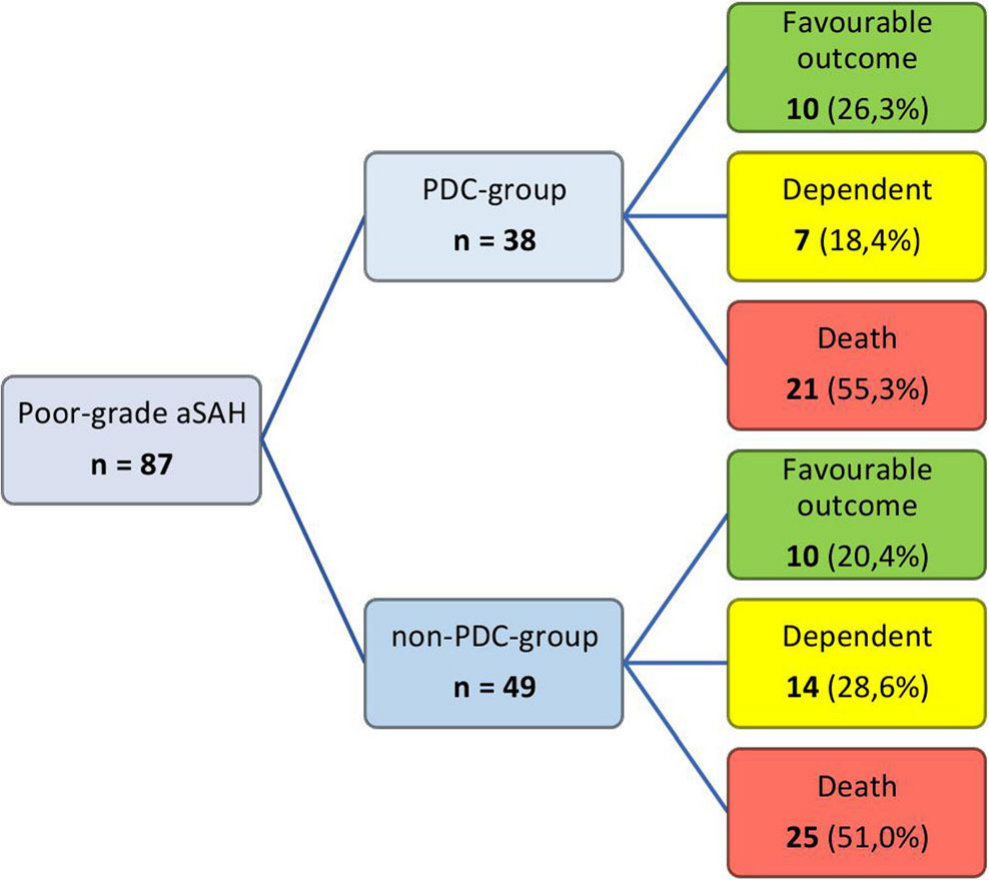
Flowchart of poor-grade aSAH patients with and without PDC and their long-term neurological outcome after 2 years

Between October 2012 and August 2017, a total number of 87 consecutive poor-grade (WFNS grade IV and V) aSAH patients were treated at our institution (Fig. 1). All of these treated patients were included for further analysis. Patients were excluded if they presented as WFNS grades I–III on admission. The diagnosis of SAH was confirmed by emergency computed tomography (CT) scanning and additional CT-angiography (CT-A). All patients underwent digital subtraction angiography (DSA) immediately after stabilization. In each case, the treatment decision (clipping or coiling) was based on an interdisciplinary approach.

PDC was performed according to the decision of the officiating neurovascular neurosurgeon. Decision-making was based on clinical signs of herniation or brain swelling, presence of large intracerebral hematoma (ICH), and/or intraoperative evidence of brain swelling [6, 18, 27].

Eventually, PDC was performed in 38 patients (PDC group)—either simultaneously within aneurysm clipping procedure or, in 2 cases, immediately after endovascular treatment.

Included for the control group (non-PDC group) were all other poor-grade aSAH patients treated in the same period of time, who did not undergo PDC (49 patients). A secondary decompressive craniectomy in this group was performed in case of treatment for refractory-elevated intracranial pressure (ICP) as ultima ratio therapy.

Best medical treatment according to the current guidelines [7, 31] was targeted in both treatment groups. Time of mechanical ventilation and length of stay on intensive care unit (ICU) were collected routinely.

### Decompressive craniectomy and intensive care unit therapy management

**Fig. 2:**
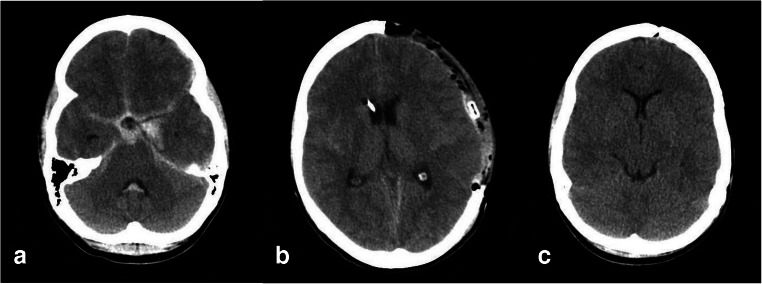
CT scans of a 21-year-old WFNS grade 5 aSAH-patient: preoperative scan (**a**), 24 h-scan after clipping and PDC (**b**), and after cranioplasty 3 months later (**c**)

DC was performed by removing a large (at least 12 × 15 cm) fronto-temporo-parieto-occipital bone flap. The temporal bone was removed osteoclastically down to the base of the middle fossa, and the dura was opened widely in a stellate fashion to minimize dural tension as described before [14]. The bone flap was cryoconserved at − 80 °C, and cranioplasty was performed approximately 3 months after DC. Exemplary CT scans are shown in Fig. 2.

After DC, all of the patients were transferred to ICU for postoperative therapy. To assess procedural complications, CT scan was performed routinely 24–36 h after aneurysm surgery or endovascular aneurysm treatment. ICP was monitored with external ventricular drain (EVD) or intraparenchymal ICP probe (Spiegelberg). Increased ICP was treated with intermittent cerebrospinal fluid (CSF) drainage, mild hyperventilation, and osmotherapy. Nimodipine was administered for 21 days after ictus (6 × 60 mg/day); transcranial Doppler (TCD) ultrasound was performed daily in all patients. At patients who underwent aneurysm clipping, a routinely postoperative DSA was performed 7–10 days after the surgery.

### Outcome measurements

The patients underwent routine follow-up, and outcome was assessed according to the modified Rankin Scale (mRS) at 6 months, at 1 year, and at 2 years post-hemorrhage. For further analysis, outcome was dichotomized into favorable (mRS 0–3) versus unfavorable (mRS 4–6) outcome according to previous trials on the effect of decompressive craniectomy in the course of malignant infarction [20].

### Matched-pair analysis

To exclude confounding factors with impact on outcome [20], we performed a matched-pair analysis. Matching parameters were age, admission status (according to WFNS grade), Fisher scale, and the presence of an additional intraventricular and/or intracerebral hematoma (IVH or ICH) (Table 3).

### Statistics

Patient characteristics including size, localization, and treatment of aneurysm; presence of intracerebral hematoma (ICH); signs of cerebral herniation (i.e., anisocoria and bilateral pupil dilatation); radiological features; use of anticoagulation or antiplatelet drugs; and functional outcome were prospectively entered in an SPSS database (version 24.0, IBM Corp., Armonk, NY). Statistical analyses were performed with an unpaired *t* test for continuous variables; categorical variables were analyzed in contingency tables using the Fisher’s exact test. Adjusted odds ratio (OR) and 95% confidence interval (CI) were calculated. Results with *p* < 0.05 were considered statistically significant.

### Systematic literature review

A search was designed to identify relevant articles on PDC in poor-grade aSAH. A comprehensive literature search was performed on MEDLINE (PubMed) database up to August 2019. The medical subject headings were (used in combination with “subarachnoid hemorrhage” and “aneurysmal subarachnoid hemorrhage”) “decompressive craniectomy,” “hemicraniectomy,” and “decompression + craniectomy.” One author (S.B.) screened title and abstract from the search results and scrutinized the references of the included publications for additional studies. Two authors (S.B. and E.G.) independently read full text of eligible studies and a consensus on which studies to include was reached after discussion among the two authors (S.B. and E.G.) considering the following inclusion and exclusion criteria.

**Fig. 3:**
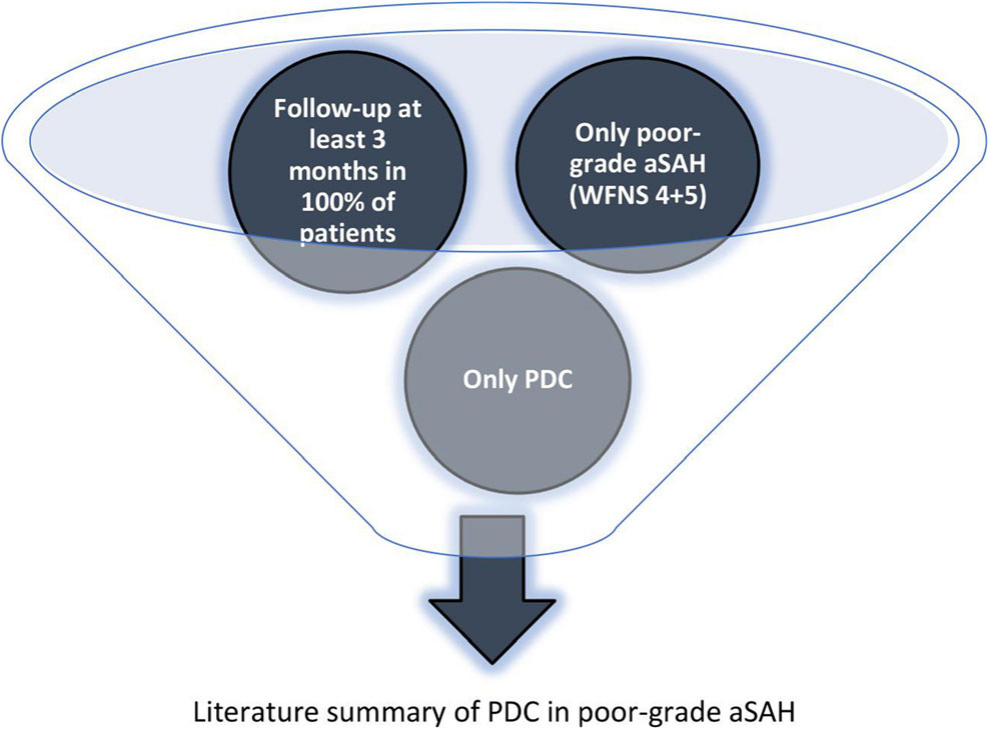
Including criteria for literature summary

In order to clarify the effect of an early decompressive craniectomy (PDC) on functional outcome in poor-grade aSAH patients, only studies have been included, in which the following individual patient data could be extracted out of the original publication: (1) Patients with WFNS or Hunt and Hess grade IV or V on admission underwent an early decompressive craniectomy (mainly < 24 h after ictus). (2) Functional outcome data had to be collected in 100% of the patients (except death). (3) The earliest captured outcome had to be 3 months after hemorrhage. The including criteria are summarized in Fig. 3.

In case of multiple publications from the same author/institution on the same topic in the same period of time, the most relevant article was elected in order to avoid inclusion of overlapping patients. Outcome was assessed by modified Rankin Scale (mRS), Glasgow Outcome Scale (GOS), or extended Glasgow Outcome Scale (GOSE). We dichotomized functional outcome as favorable (mRS 1–3, GOS 4–5, and GOSE 5–8) versus unfavorable (mRS 4–6, GOS 1–3, and GOSE 1–4). In every study included, the context between WFNS (or Hunt and Hess) grade on admission, PDC as treatment modality, and long-term neurological outcome had to be extractable.

Studies, in which the authors could not extract these detailed patient data from the original publication, were excluded from the analysis. The other exclusion criteria were the following: single case reports, conference abstracts, and studies including non-aneurysmal SAH.

**Fig. 4:**
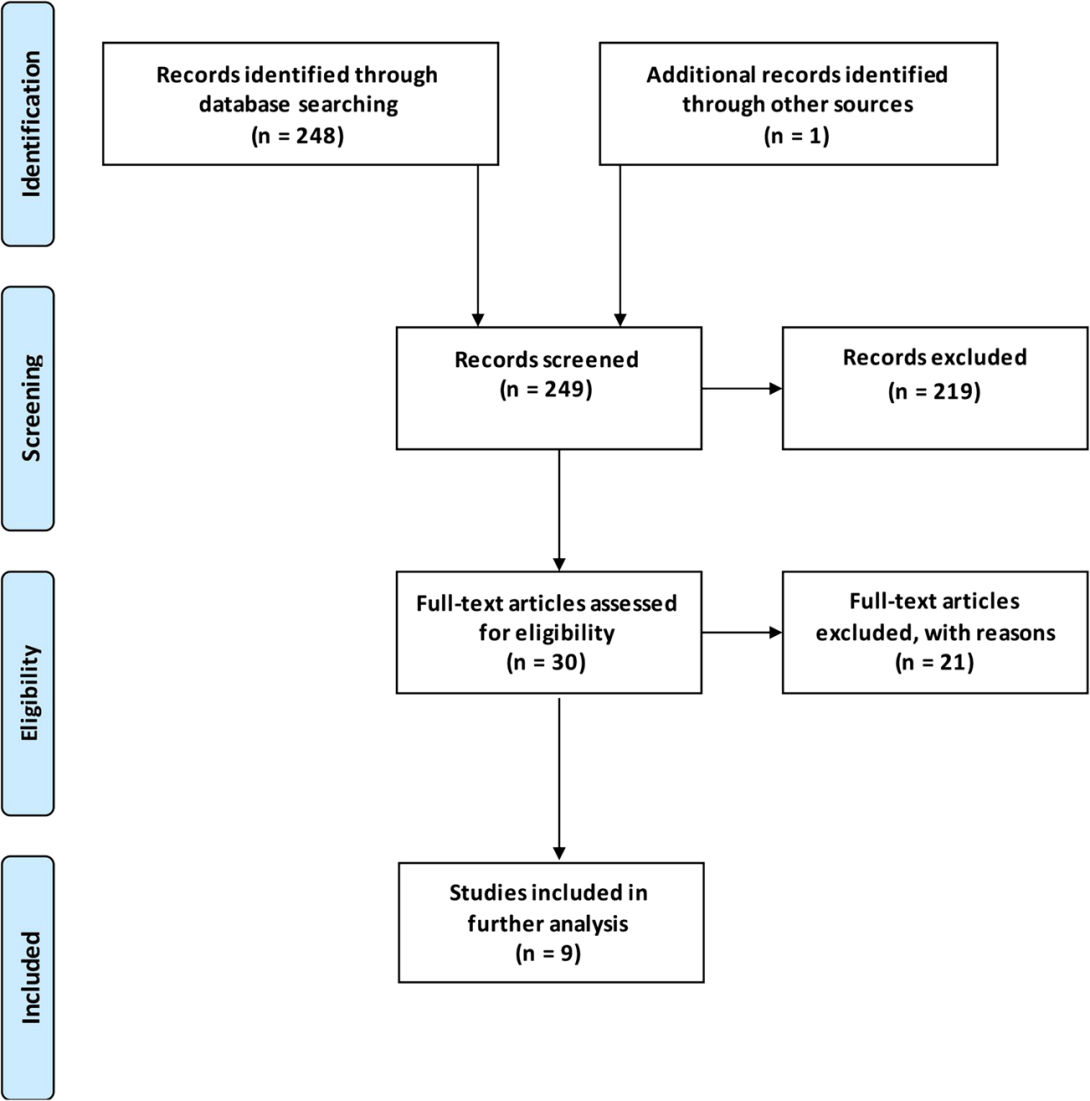
Flowchart depicting the search strategy of systematic literature review

A flowchart depicting the search strategy of systematic literature review is shown in Fig. 4.

## Results

### Patient characteristics

Between October 2012 and August 2017, 38 patients with poor-grade aSAH underwent PDC (PDC group). Mean age was 54 years (± 12 years), among them 28 female patients (74%). During the same period, 49 patients with poor-grade aSAH did not undergo PDC (non-PDC group). Mean age was 61 years (± 11 years), among them 34 female patients (69%). The baseline characteristics of the patients of both groups are given in Table 1. Microsurgical clipping was performed in 36 of 38 patients (95%) in the PDC group compared with 8 of 49 patients (16%) in the non-PDC group (*p* < 0.0001).

**Table 1:** Baseline characteristics of 87 poor-grade aSAH patients with or without PDC

*Characteristics, n (%)*	PDC group	non-PDC group	*p* value
*No. (male/female)*	38 (10/28)	49 (15/34)	*0.812*
*Mean age (years) (± SD)*	54 (± 12)	61 (± 11)	*0.006*
*WFNS grade V (%)*	33 (87)	32 (65)	*0.026*
*Fisher grade III–IV (%)*	38 (100)	49 (100)	*1.000*
*Mean aneurysm size in mm (± SD)*	12 (± 8)	8 (± 6)	*0.009*
*Multiple aneurysms (%)*	9 (24)	12 (24)	*1.000*
*Clipping (%)*	36 (95)	8 (16)	*< 0.0001*
*Posterior circulation aneurysm (%)*	0 (0)	17 (35)	*< 0.0001*
*ICH (%)*	26 (68)	13 (27)	*0.0002*
*Dilated pupil (%)*	22 (58)	13 (27)	*0.0042*
*SDC (%)*	0 (0)	14 (29)	*0.0002*
*Anticoagulation/antiplatelet drugs (%)*	9 (24)	18 (37)	*0.245*
*Hypertension (%)*	13 (34)	20 (41)	*0.657*
*Hospital stay in d (± SD)*	34 (± 32)	33 (± 28)	*0.877*
*Ventilation in h (± SD)*	387 (± 376)	385 (± 367)	*0.980*
*Favorable mRS at 6-month follow-up (%)*	7 (18)	10 (20)	*1.000*
*Favorable mRS at 1*-*year follow-up (%)*	9 (24)	10 (20)	*0.796*
*Favorable mRS at 2*-*year follow-up (%)*	10 (26)	10 (20)	*0.610*
*Mortality after 2 years (%)*	21 (55)	25 (51)	*0.829*

*ICH* intracerebral hemorrhage, *mRS* modified Rankin Scale, *(P)/(s) DC* (primary)/(secondary) decompressive craniectomy, *WFNS* World Federation of Neurosurgical Societies; *p* < 0,05 considered statistically significant

A secondary decompressive craniectomy (SDC) in the non-PDC group was performed in 14 patients (29%) in case of treatment for refractory elevated ICP as ultima ratio therapy.

### Patients in the PDC group had a significantly worse clinical status at presentation

Twenty-six of thirty-eight patients (68%) in the PDC group were presenting with additional ICH compared with thirteen of forty-nine patients (27%) in the non-PDC group (*p* = 0.0002). Twenty-two of thirty-eight patients (58%) in the PDC group had clinical signs of cerebral herniation at presentation (uni- or bilateral dilated pupil), compared with thirteen of forty-nine patients (27%) in the non-PDC group (*p* = 0.0042). Thirty-three of thirty-eight patients (87%) in the PDC group presented as WFNS grade 5 on admission compared with thirty-two of forty-nine patients (65%) in the non-PDC group (*p* = 0.03).

The most frequent localization of the ruptured aneurysm in the PDC group was the middle cerebral artery (MCA, 42%), followed by the internal carotid artery (ICA, 26%). In the non-PDC group, the most frequent localization was the anterior communicating artery (ACoA, 35%). Patients treated with PDC had significantly larger aneurysms (mean size 12 mm (± 8 mm) versus 8 mm (± 6 mm); *p* = 0.01) assessed by DSA. There were three giant aneurysms (≥ 25 mm) in PDC group (8%) and two in the non-PDC group (4%); *p* = 0.65. Multi aneurysms were found in 24% of the patients in each group (*p* = 1.00).

Prior use of anticoagulation/antiplatelet drugs and high blood pressure as pre-existing condition did not differ between the two groups: Nine of thirty-eight patients (24%) in the PDC group were on anticoagulation/antiplatelet therapy prior to ictus compared with eighteen of forty-nine patients (37%) in the non-PDC group (*p* = 0.25). Pre-existing high blood pressure was diagnosed in thirteen of thirty-eight patients (34%) versus twenty of forty-nine patients (41%) (*p* = 0.66).

The duration of mechanical ventilation and the duration of hospitalization did not differ between the groups **(**Table 1**)**.

### Neurological outcome

Outcome measures were assessed at 6 months, at 1 year, and at 2 years after aSAH. Follow-up data was available for all patients. Overall, after 2 years, 10 of 38 (26%) patients who underwent PDC achieved a favorable outcome compared with 10 of 49 (20%) patients in the control group (*p* = 0.61).

Mortality rate after 2 years did not differ between the two groups: 55% after PDC versus 51% in patients without PDC (*p* = 0.83). Outcome and mortality rates after 2 years of all patients are shown in Table 2**.**

**Table 2:** Patient neurological outcome (mRS) and mortality after 2 years in single-center cohort

		All patients (%) *n* = 87	PDC group (%) *n* = 38	non-PDC group (%) *n* = 49
Favorable	mRS 0	3 (3.4)	2 (5.3)	1 (2.0)
	mRS 1	5 (5.7)	2 (5.3)	3 (6.1)
	mRS 2	9 (10.3)	4 (10.5)	5 (10.2)
	mRS 3	3 (3.4)	2 (5.3)	1 (2.0)
Unfavorable	mRS 4	10 (11.5)	4 (10.5)	6 (12.2)
	mRS 5	11 (12.6)	3 (7.9)	8 (16.3)
	mRS 6	46 (52.9)	21 (55.3)	25 (51.0)
	Fav. outcome (mRS 0–3)	20 (23.0)	10 (26.3)	10 (20.4)
	mRS 0–4	30 (34.5)	14 (36.8)	16 (32.7)
	Mortality	46 (52.9)	21 (55.3)	25 (51.0)

*mRS* modified Rankin Scale, *(P)DC* (primary) decompressive craniectomy

### Matched-pair analysis

In a matched-pair analysis of 38 patients (PDC group) versus 25 patients (non-PDC group), initial clinical status at presentation did not differ significantly (baseline characteristics and outcome are shown in Table 3). While initial status did not differ between the two groups, there was a trend towards better long-term neurological outcome in the PDC group compared with the non-PDC group (*p* = 0.1).

**Table 3:** Baseline characteristics and outcome in matched-pair analysis

*Characteristics, n (%)*	PDC group	non-PDC group	*p* value
*No. (male/female)*	38 (10/28)	25 (6/19)	*1.00*
*WFNS grade V (%)*	33 (87)	18 (72)	*0.19*
*Fisher grade III–IV (%)*	38 (100)	25 (100)	*1.00*
*ICH (%)*	26 (68)	13 (52)	*0.29*
*Dilated pupil (%)*	22 (58)	9 (36)	*0.12*
*Favorable mRS at 2*-*year follow-up (%)*	10 (26)	2 (8)	*0.10*
*Mortality after 2 years (%)*	21 (55)	15 (60)	*0.80*

*ICH* intracerebral hemorrhage, *mRS* modified Rankin Scale, *(P)DC* (primary) decompressive craniectomy, *WFNS* World Federation of Neurosurgical Societies; *p* < 0,05 considered statistically significant

### Systematic literature review

The search strategy is shown in Fig. 4. The initial MEDLINE search yielded a number of 248 studies. There was one additional record through checking the reference lists of the included studies. Of the 248 records screened, 29 full-text articles were assessed for eligibility, 9 of which met the inclusion criteria [3, 6, 8, 21, 22, 26, 27, 30, 38]. Data of the 9 included studies are summarized in Table 4. The 9 included studies contained a total number of 165 DC patients in aSAH. Extracting the cases of PDC in poor-grade aSAH patients, a patient number of 131 left over. Mean age of this patient group was 53 years (SD 5.66) and the mean follow-up time was 11 months (SD 3.71). In total, nearly half of the patients (64 of 131 patients (49%)) achieved a favorable functional long-term outcome.

**Table 4:** Summary of 9 studies after systematic review of the literature for PDC in poor-grade aSAH

Authors and year	DC patients (*n*)	Hunt and Hess 4 + 5 (%)	PDC in poor-grade aSAH (*n*)	Mean age of PDC group (years)	Mean follow-up (months)	Mortality of PDC group (%)	Favorable outcome in PDC patients* (%)
Arikan et al., 2010	11	100	11	45	12	45 (5/11)	36 (4/11)
Buschmann et al., 2007	38	82	18	50	12	33 (6/18)	44 (8/18)
D’Ambrosio et al., 2005	12	100	8	58	12	13 (1/8)	63 (5/8)
Kazumata et al., 2010	11	100	11	60	12	18 (2/11)	36 (4/11)
Lu et al., 2014	26	100	26	44	6	19 (5/26)	42 (11/26)
Russegger et al., 1993	19	100	19	57	≥ 3	21 (4/19)	47 (9/19)
Schirmer et al., 2007	16	69	6	51	15	17 (1/6)	67 (4/6)
Smith et al., 2002	8	100	8	56	12	13 (1/8)	75 (6/8)
Zhao et al., 2015	24	100	24	52	12	29 (7/24)	58 (14/24)
TOTAL	165		131	53	Median: 11	24 (32/131)	49 (64/131)

*Favorable outcome: modified Rankin Scale (mRS) scores 0–3; Glasgow Outcome Scale (GOS) scores 4 and 5; extended Glasgow Outcome Scale (GOSE) scores 5–8; and classification as “fair”, “good”, and “excellent”

## Discussion

### Properties of PDC

Patients with poor-grade aSAH often have poor prognosis with high case fatality and disability rates [4, 7, 23, 37]. The effect of DC in this selected subset of patients was reported in multiple studies [3, 6, 8, 9, 12, 13, 16–19, 21, 22, 24–27, 30, 33, 34, 36, 38]. An early decompression (PDC) may intensify the protective properties of DC: reducing elevated ICP immediately after bone flap removal and dura opening and furthermore leading to an improvement in tissue perfusion and oxygenation. The question of indication and right timing for decompressive craniectomy became subject of a controversial debate. In 2009, Güresir et al. [13] published a large cohort of 79 patients with decompressive craniectomy in subarachnoid hemorrhage. Their results indicated that DC might be warranted regardless of whether the patient suffers from bleeding, infarction, or brain swelling. The authors stated that the best time for DC must still be defined, but an early decompression seems to be beneficial, leading to favorable outcome in 25.3% of their patients.

### Encouraging outcome data after PDC

Beside an approved life-saving effect, it is still debatable if an early decompression provides a better long-term functional outcome with lower rates of severe disability and dependency rates of patients.

In our single-center analysis of poor-grade aSAH patients, we compared the neurosurgical treatment course with and without performing PDC (Table 1); patients in the PDC group had a significantly worse clinical status at presentation (significant higher rate of mydriasis/cerebral herniation and additional ICH and significant higher number of WFNS grade 5 classified patients). This led to the assumption that these patients achieve worse neurological long-term outcome, but in fact, favorable outcome at 2 years post-hemorrhage did not differ significantly between the two groups.

While historically these severely affected patients did not undergo any further treatment due to high mortality rates, our data reveals encouraging results of long-term outcome in these patients.

### Matched-pair analysis

In a matched-pair analysis of 38 patients (PDC group) versus 25 patients (non-PDC group), where initial clinical status (WFNS grade, ICH, dilated pupil(s)/cerebral herniation) was equally distributed, results of favorable long-term neurological outcome reveal a trend towards a higher rate of favorable neurological outcome in the PDC group compared with the non-PDC group (*p* = 0.1). However, the patient cohort was too small to draw definitive conclusions. Baseline characteristics and outcome are shown in Table 3.

### PDC in literature review

In the present systematic literature review, most of the authors came to the conclusion that aSAH patients who underwent an early DC seem to benefit the most regarding their long-term functional outcome. In 1993, Russegger et al. [26] recommended a “peracute aneurysm surgery,” defined as that the operation has to be performed “immediately after hospitalization or diagnosis.” All of their nineteen poor-grade aSAH patients underwent PDC immediately after admission (between 2 and 8 h after bleeding onset). Nine of the nineteen patients (47%) reached a favorable outcome (defined as “fair,” “good,” or “excellent”) after 1 year. Smith et al. [30] emphasized the potentially beneficial effect on the control of local and global ICP gradients and even proposed a prophylactic DC for the treatment of poor-grade aSAH patients with ICH. Seventy-five percent of their patients (6 out of 8) achieved a favorable outcome (“fair,” “good,” or “excellent”) after 1 year, which is the highest rate of favorable outcome in the included studies. In D’Ambrosio et al. [8], the study group included 12 poor-grade aSAH patients who underwent DC. Excluded of meta-analysis had been 4 patients, who “returned to operating room for hemicraniectomy after initial aneurysm repair operation.” Out of 8 PDC patients, 5 achieved a favorable outcome after 1 year (62.5%; GOS 4 + 5), and they concluded that DC may be indicated “if performed early and in a specific subset of patients.”

Buschmann et al. [6] presented 38 aSAH patients who underwent DC and summarized that patients “with progressive brain edema without radiological signs of infarctions and those with hematoma and consecutive ICP elevations might benefit most from DHC.” For our data analysis, we extracted the 21 patients in “group 1: primary DHC,” 18 of which were classified as poor grade on admission. Of these 18 patients, favorable outcome (GOS 4 + 5) was achieved in 44%. Schirmer et al. [27] stated that patients seem to have a better outcome, “in whom early decompression was performed within 48 hours after aneurysm rupture.” They published a cohort of 16 patients who underwent DC, 11 of which were presenting as poor grade on admission. Of these 11 poor-grade patients, 6 underwent PDC (defined as “time to craniectomy”: *d* = 0 + *d* = 1), and 4 of them achieved a favorable outcome (67%; mRS 0–3). Remarkably, Schirmer et al. showed that DC as treatment modality could significantly decrease refractory intracranial hypertension even in the absence of large intraparenchymal hemorrhage.

Furthermore, we could extract patient data about 11 poor-grade aSAH patients from Kazumata et al. [21]. Of these 11 patients who underwent an early external decompression, 4 patients achieved a favorable outcome (36%; GOSE 5–8). Confirming our hypothesis, Kazumata et al. stated that an aggressive decompression in “acute stage may prevent significant postoperative brain swelling.” From the studies of Lu et al. [22], Arikan et al. [3], and Zhao et al. [38], no separate patient data was needed to be extracted. In both studies, only cases of PDC in poor-grade aSAH patients were presented with rates of a favorable outcome of 42% (Lu et al. [22]), 36% (Arikan et al. [3]), and even 58% (Zhao et al. [38]). Moreover, Zhao et al. reported about their control group without DC, in which overall 57% of the patients achieved a favorable outcome, which lead the author to the statement that PDC “does not seem to be significantly associated with improved outcomes.”

Beside these, Jussen et al. [19] published total number 11 PDC patients (DC in mean 2.6 ± 1.4 h after admission), of which 25% made a good recovery compared with 12.5% in the secondary DC group. The author advocated an “ultra-early DC” especially for poor-grade patients with intracerebral mass lesion. Data of this publication was not included in the systematic literature review, because a long-term follow-up was not obtained in 16% of the patients.

Eventually, Jabbarli et al. [18] published the largest-to-date monocentric series of DC in aSAH. Their subgroup of PDC patients was subdivided into early (≤ 24 h after ictus) and delayed PDC. For all WFNS grade patients, a favorable outcome was achieved in 40% of the patients in the early PDC group and in 22% of the patients in the delayed PDC group. The publication has not been included in systematic literature review, because detailed patient data for the subgroup of poor-grade patients (Hunt and Hess 4 + 5; WFNS 4 + 5) could not be extracted. However, Jabbarli et al. stated that “especially SAH individuals with the admission WFNS < 5 strongly benefit from early craniectomy,” which led him to the conclusion that indication for DC should not be restricted only for poor-grade aSAH patients.

In total, out of the 131 poor-grade aSAH patients in the systematic literature review, nearly half of the patients achieved a favorable functional long-term outcome (49%).

### Advantages of PDC

Our results indicate that PDC may be warranted in this severely ill subset of patients. We believe that PDC may intensify the protective properties of DC through immediately reducing ICP, preventing secondarily ICP increase through allowing parenchymal swelling in the post-hemorrhagic period to a certain extent, thereby could avoid cerebral hypoperfusion. Furthermore, the risk of a second operation (secondary decompressive craniectomy) in the critical phase of illness is minimized. Even though there was a trend towards a better outcome for PDC patients, we also found PDC was not significantly associated with improved outcomes in our study cohort. One possible reason might be the worse initial clinical condition of patients in our PDC group (additional ICH, cerebral herniation, WFNS grade 5) compared with the PDC patients in literature research. Thus we had, for example, a high percentage of cases with cerebral herniation (58%) and WFNS grade 5 (87%), compared with other publications (e.g., Lu et al. [22]: 33% cerebral herniation and 46% WFNS grade 5).

### Study limitations

The study has several limitations. First of all, the retrospective design: Patient data in our single-center-cohort as well as in the included studies of the review of literature was acquired retrospectively. The sample size of our PDC group is quite small, but at least it is the largest PDC cohort of poor-grade aSAH patients with detailed information compared with the included studies from the literature review. The quite small number of patients limits the accuracy of the results. Furthermore, the patients have not been randomized in our cohort study—the decision-making varied according to the attending vascular neurosurgeon.

Furthermore, there are two main limits of our literature research: First, in the study populations, respectively the patient number of each study included for further analysis is small. Secondly, for most studies included, there is a lack of a consistent control group and thus a lack of comparisons demonstrating any potential advantage of PDC. One must consider that the relatively small number of patients does not justify formulating guidelines concerning indication of PDC. To draw definitive conclusion about indication and timing of DC, further prospective studies with standardized treatment protocols are needed.

However, we provide for the first time a single-center cohort analysis combined with a systematic literature review for PDC in poor-grade aSAH.

## Conclusions

According to the current data, PDC may be a valid treatment option in the treatment of poor-grade aSAH. Despite a worse clinical status at presentation (significant higher rate of mydriasis and additional ICH), poor-grade aSAH patients who underwent PDC achieve favorable outcome in a significant number of patients.

Summarizing the published cases of PDC, in nearly half of the patients, a favorable long-term functional outcome could be achieved.

An early decompression might prevent incontrollable elevated ICP and/or infarction due to vasospasm and thus may intensify the protective properties of DC through allowing parenchymal swelling in the post-hemorrhagic period to a certain extent and avoiding cerebral hypoperfusion.

Although the number of patients in this study is small, our findings suggest that PDC could lead to a better functional outcome. Therefore it should not be omitted in this severely ill subset of patients. To clarify a possible benefit of PDC on clinical outcome in poor-grade aSAH patients, a multicenter, prospective randomized controlled study (PICASSO trial—Primary decompressive craniectomy in aneurysmal subarachnoid hemorrhage, DRKS00017650) was initiated by the senior author as investigator-initiated trial.
